# Evaluating the effects of variation in clinical practice: a risk adjusted cost-effectiveness (RAC-E) analysis of acute stroke services

**DOI:** 10.1186/1472-6963-12-266

**Published:** 2012-08-21

**Authors:** Clarabelle Pham, Orla Caffrey, David Ben-Tovim, Paul Hakendorf, Maria Crotty, Jonathan Karnon

**Affiliations:** 1 Discipline of Public Health, The University of Adelaide, South Australia, Australia; 2 Health Economics Unit, University of Birmingham, Birmingham, UK; 3 Clinical Epidemiology Unit, Flinders Medical Centre, South Australia, Australia; 4 Department of Rehabilitation and Aged Care, Flinders University of South Australia, South Australia, Australia

## Abstract

**Background:**

Methods for the cost-effectiveness analysis of health technologies are now well established, but such methods may also have a useful role in the context of evaluating the effects of variation in applied clinical practice. This study illustrates a general methodology for the comparative analysis of applied clinical practice at alternative institutions – risk adjusted cost-effectiveness (RAC-E) analysis – with an application that compares acute hospital services for stroke patients admitted to the main public hospitals in South Australia.

**Methods:**

Using linked, routinely collected data on all South Australian hospital separations from July 2001 to June 2008, an analysis of the RAC-E of services provided at four metropolitan hospitals was undertaken using a decision analytic framework. Observed (plus extrapolated) and expected lifetime costs and survival were compared across patient populations, from which the relative cost-effectiveness of services provided at the different hospitals was estimated.

**Results:**

Unadjusted results showed that at one hospital patients incurred fewer costs and gained more life years than at the other hospitals (i.e. it was the dominant hospital). After risk adjustment, the cost minimizing hospital incurred the lowest costs, but with fewer life-years gained than one other hospital. The mean incremental cost per life-year gained of services provided at the most effective hospital was under $20,000, with an associated 65% probability of being cost-effective at a $50,000 per life year monetary threshold.

**Conclusions:**

RAC-E analyses can be used to identify important variation in the costs and outcomes associated with clinical practice at alternative institutions. Such data provides an impetus for further investigation to identify specific areas of variation, which may then inform the dissemination of best practice service delivery and organisation.

## Background

Evidence-based clinical guidelines have been developed for the treatment of acute stroke events, which define recommended processes for diagnosing, treating, and monitoring stroke [1]. However, clinical practitioners are well aware that the dissemination of guidelines does not prevent the emergence of differences in practices between clinical services. The dilemma is how to evaluate the outcome of the day-to-day practices of different services in such a way as to identify the areas of variations that have long-term benefits for patient care. This is particularly important when reducing variation requires resources over and above currently allocated budgets, but the benefits to patients accrue over time, without being identifiable at the point of discharge from hospital. Evaluating service differences as investments rather than costs is a constant source of friction between health practitioners and the funders of health services.

Cost-effectiveness analysis is now well established in relation to pharmaceuticals and medical technology, but not in relation to broader institutional level variations in day-to-day clinical practice. This precludes the identification of efficient practice, and the establishment of processes to guide and finance investment in service delivery and organisation (as are established in many countries for specific health care technologies). The gap in the application of cost-effectiveness analysis is likely due to the complex nature of evaluating alternative forms of routine clinical practice.

Linked routinely collected data provides a potentially useful data source for the evaluation of clinical practice, with the benefits of rapid and inexpensive access to large amounts of data that describes real world activity. Internationally, systems are currently being developed to increase availability and access to linked routinely collected data. We have developed a novel methodology that uses linked routinely collected data to evaluate the long-term costs and benefits of services for specific conditions provided at alternative hospitals (risk adjusted cost-effectiveness (RAC-E) analysis) [2,3]. This paper reports an application of the RAC-E methodology to evaluate the relative cost-effectiveness of acute stroke services at the four main public hospitals in South Australia (SA).

## Methods

**Figure 1:**
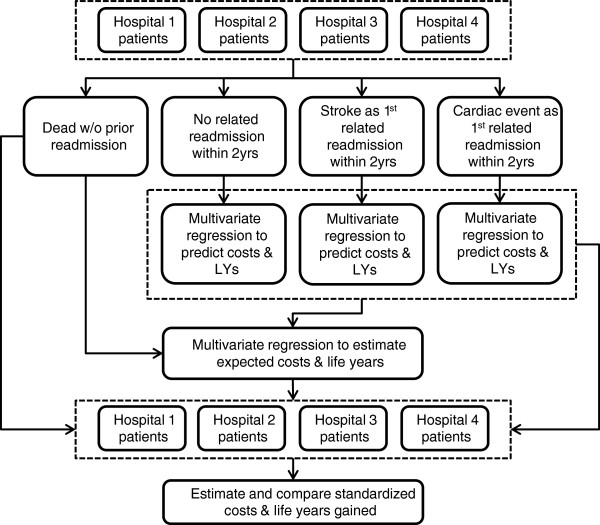
RAC-E analytic framework.

Figure 1 illustrates the RAC-E analytic framework. In the first stage, all eligible patients with an initial stroke event over an extended time horizon (from July 2002 to June 2008) are assigned to one of four mutually exclusive intermediate endpoints representing the first (if any) event of interest. Separate datasets for the ‘no related readmission nor death’, ‘non-fatal recurrent stroke’ and ‘non-fatal cardiac event’ cohorts are created.

Using the three datasets, separate estimates of overall survival from the time of their intermediate endpoint are estimated for each patient by fitting parametric survival curves to the observed data. Long-term costs are generated alongside the parametric survival models, using multivariate regression models to predict annual cost estimates.

The ‘observed + extrapolated’ cost and survival estimates for the cohort of interest (patients with a new stroke between July 2005 and June 2006) across the four intermediate endpoints are then combined into a single dataset. This dataset is analyzed using multivariate regression models to generate estimates of expected costs and survival for each patient, from the time of their initial stroke separation.

Combining the above data for all patients with a stroke separation between July 2005 and June 2006, risk adjusted cost and survival estimates are generated as the ‘observed + extrapolated’ values minus the corresponding expected values for each patient. Mean risk adjusted cost and survival estimates are then generated for each hospital, which inform estimates of incremental cost-effectiveness between the clinical processes at the alternative institutions.

### Data sources

Routinely collected data was obtained and linked from the following sources:

● Hospital separation data 4,072,341 records from the Integrated South Australian Activity Collection (ISAAC), describing patient, admission, and inpatient stay characteristics for all hospital separations in South Australia from July 2001 to June 2008. Co-morbidity variables were coded based on the current clinical performance indicators for the Variable Life Adjusted Display (VLAD) system using ICD-10.5-AM codes [4].

● Socioeconomic data Area (postcode) level variables describing socioeconomic areas, socioeconomic disadvantage, economic resources, and education and occupation.

● Costing data 1,530,634 separation-specific cost estimates at the four largest hospitals in South Australia from July 2003 to June 2008, presented in 16 categories covering direct and indirect ward, surgery, allied health, diagnostics, pharmacy, and prostheses related costs.

● All-cause mortality data 92,288 deaths from the Register for Births, Deaths, and Marriages between July 2001 and December 2008.

The separations within the ISAAC data were linked deterministically using an algorithm that specified thresholds for positive linkages over a set of common identifiers [5,6]. The costing and socioeconomic data were matched with the ISAAC data on the basis of hospital-specific identification numbers and postcodes, respectively. The mortality and ISAAC data were linked probabilistically using mathematical algorithms to determine the likelihood that a pair of records refers to the same individual given a set of common identifiers. Extensive clerical review was also undertaken to confirm the linkages between the mortality and ISAAC data.

Ethics approval was granted by the South Australian Department of Health Human Research Ethics Committee (HREC Protocol No.: 264/11/2011).

### Study cohort

Stroke separations may be identified via ICD-10.5-AM principal diagnosis codes and AR-DRG codes. Inclusion criteria for the study included ICD principal diagnosis codes for stroke (I60-I64) and an AR-DRG stroke code (B70), which includes sub-categories that differentiate according to the presence of catastrophic (B70A), severe (B70B), and neither catastrophic or severe (B70C) comorbidities or complications. Other AR-DRG codes with a principal diagnosis of stroke included craniotomy (B02), extracranial vascular procedure (B04) and tracheostomy or ventilation >95 hours (A06), which were excluded in order to increase the homogeneity of the defined patient cohort. Other exclusion criteria included deaths within 5 days of admission, which was assumed to be outside the influence of acute hospital care.

The main study cohort comprised patients admitted to one of the four study hospitals with a B70 AR-DRG code in the year to June 30, 2006, excluding patients with a documented stroke diagnosis in the year prior to this date. Using the Brameld-Holman backcasting method [7] to address the prevalent pool effect, it was determined that this cut-off date provided a 90% probability that the first observed stroke was a new stroke.

### Extrapolation analysis

To inform the extrapolated analysis of lifetime costs and survival, all patients with a new stroke admission (as defined for the study cohort) from July 1, 2002 were allocated to one of the four intermediate endpoints: non-fatal recurrent stroke; non-fatal major cardiac event; death without, or within 28 days of a non-fatal stroke or major cardiac event; or no subsequent event. These data provided follow-up data of up to 6 years to inform the extrapolation analyses, i.e. follow-up data on all patients experiencing an intermediate event were available to June 2008, unless they died in the interim period.

Non-fatal recurrent stroke was defined as any hospital admission with an ICD principal diagnosis of stroke. Based on an analysis of survival of stroke patients following any readmission within Major Disease Category 5 (Diseases of the Circulatory System), twenty DRG codes with a mean 1-year mortality rate of 40% or greater were defined as non-fatal major cardiac events. Both non-fatal events excluded patients who died within 28 days of admission, and hospital transfers from the index stroke admission.

The following sections describe the methods used to extrapolate lifetime costs and survival beyond each intermediate endpoint.

### Survival models

Flexible parametric models for survival analysis, introduced by Royston and Parmar [8], were applied to the three intermediate endpoint datasets (containing patients experiencing a non-fatal stroke, non-fatal cardiac event, or no subsequent event, respectively) to predict survival beyond each intermediate endpoint. Every patient in the datasets had a recorded date of death, or they were censored at the end of the follow-up period (30 June 2008). Explanatory variables were based on patient clinical and socio-demographic characteristics at the time of their intermediate event (as described in the data section).

The applied survival models used restricted cubic splines to estimate log cumulative hazards, controlling for the effect of relevant patient characteristics. This is a general approach for adapting linear methods to model non-linear relationships through the use of multiple points of inflexion (knots) across the modeled time horizon [9].

To fit the models, we used backwards stepwise selection using the full range of demographic, socioeconomic, and clinical explanatory variables. The criterion for inclusion in the model was p ≤ 0.05. These initially defined models were then expanded to test for significant interactions between the included explanatory variables. Interaction terms were included in the models if they improved model fit, as judged by the Akaike's Information Criterion. The final stage of the analysis tested the effect of alternative functional forms by comparing models that fitted a restricted cubic spline with between 1 and 5 knots. To assess the overall fit of the parametric survival models, the mean survival curve was plotted against the Kaplan-Meier survival curve.

The resulting survival functions were used to estimate annual age-specific probabilities of survival, conditional on surviving to each starting age. For each patient, these probabilities were used to estimate a mean survival time between patients’ age at the time of their intermediate endpoint event and age 100 years. A 5% discount rate was applied to the survival estimates, in accordance with the Pharmaceutical Benefits Advisory Committee guidelines [10].

### Cost models

The cost data within the three intermediate endpoint datasets were formatted to estimate annual cost variables for each full year of life between patients’ intermediate endpoint and their recorded date of death or the end of the follow-up period (30 June 2008). Costs associated with any stroke or cardiac related hospital admissions were included.

Using the same explanatory variables as used in the survival models, annual costs were estimated using a two-stage process that estimated the probability of patients incurring any costs (using logistic regression), followed by an estimate of the magnitude of the cost, if incurred (using generalized linear models - GLMs). Separate models were fitted for each of the three intermediate endpoint datasets, with additional models that differentiated between costs incurred in the first year post-event and in subsequent years for the non-fatal stroke and non-fatal cardiac event endpoints [11].

Similar model selection criteria to those used for the survival models were applied. For the logistic regression analyses, overall model fit was established using the Ramsey RESET test. For each GLM, the modified Park test was used to determine the most appropriate distribution, and the appropriate link function was selected by testing different power functions with respect to the Pearson correlation, Pregibon link, and the Modified Hosmer-Lemeshow tests.

For each patient, the predicted annual cost estimates were combined with the annual survival probabilities generated from the survival models to estimate mean lifetime costs, i.e. the product of the discounted annual survival probabilities and corresponding annual cost estimates were summed across the time period between their intermediate endpoint event and age 100 years.

### Observed lifetime costs and survival

From the index stroke event, lifetime cost and survival estimates for each patient were generated, given their experienced intermediate endpoint (non-fatal stroke; non-fatal major cardiac event; no subsequent event; or death). These estimates combined observed costs and survival during the period between the index stroke event and each patient’s intermediate endpoint, with extrapolated costs and survival time beyond the intermediate endpoint (other than for patients who died during the follow-up period, for whom only observed costs and survival were used).

Despite incorporating predicted values, these cost and survival estimates are labeled as ‘observed’ values because they reflect the effects of the observed intermediate endpoints.

### Expected lifetime costs and survival

The observed lifetime costs and survival estimates for all eligible patients (i.e. across all four intermediate endpoints, for patients with a new stroke in the year to June 30, 2006) were combined into a single dataset. A GLM and a flexible parametric survival model [8] were then fitted to generate patient-specific estimates of *expected* lifetime costs and survival, respectively. Explanatory clinical and socio-demographic variables for these models reflected patient characteristics at the time of the index event (i.e. not controlling for the observed intermediate events).

### Main data analysis

Mean estimates of observed costs and survival across all patients treated at each hospital were derived, informing unadjusted estimates of the cost-effectiveness of services provided at the alternative hospitals.

Risk adjusted cost and survival estimates for each patient were generated as the relevant observed minus expected values, which provide a more intuitive interpretation of hospital performance than ‘observed divided by expected’ adjusted values.

Differences in the mean ‘observed minus expected’ cost and survival estimates between hospitals can be interpreted as risk adjusted differences in costs and survival: if costs incurred by patients at hospital A are $300 more than expected, whilst costs incurred by patients at hospital B are $200 less than expected, then the risk adjusted difference in per patient costs between hospitals A and B is $500. Revised incremental cost-effectiveness ratios were generated using these risk adjusted values.

A probabilistic sensitivity analysis was informed by a multi-stage bootstrapping (sampling with replacement) process, which precluded the need to parameterize the correlation between lifetime costs and survival. Firstly, the datasets informing each of the extrapolation regression models (10 cost models and 3 survival models) were bootstrapped, and the corresponding model coefficients re-estimated to generate new estimates of lifetime costs and survival for each eligible patient. The resulting dataset of ‘observed’ lifetime costs and survival was also bootstrapped and the coefficients for the expected costs and survival regression models re-estimated. This sequential bootstrapping process was repeated for 2,000 iterations, and the resulting outputs were used to plot cost-effectiveness acceptability curves that display the probability that each hospital is cost-effective at different threshold values for gaining additional life years.

All analyses were undertaken using Stata, release 11.0 (StataCorp LP).

## Results

Key patient characteristics for the eligible cohort of stroke patients presenting in the year to June 30 2006 are presented in Table 1. The only significant variation in patient casemix concerned socioeconomic disadvantage (p < 0.001), with patients at hospital D classified as the most disadvantaged.

**Table 1:** Stroke patient characteristics by hospital

	**Hospital A**	**Hospital B**	**Hospital C**	**Hospital D**	**P-value**
**Age** (mean (SD), years)	75.9 (11.4)	76.2 (10.4)	73.3 (12.6)	72.0 (14.3)	0.003
**Sex** (% male)	51	46	54	51	NS
**Stroke severity** (%):					0.001
B70A (Stroke + CCC)	29	46	31	30	
B70B (Stroke + SCC)	38	34	35	43	
B70C (Stroke -CSCC)	33	20	34	27	
**Intermediate outcome** (% patients):					0.063
No event	64	57	54	53	
Recurrent stroke	6	5	5	9	
Major cardiac event	7	6	13	9	
Death within 2 years	23	32	28	29	
**Co-morbidities** (% patients):					
Other COPD	7	12	6	12	0.045
Anaemia	8	15	10	12	NS
Diabetes mellitus	24	31	19	22	0.036
Acute LRTI & influenza	10	11	10	8	NS
UTI	11	28	22	15	<0.001
Other urinary symptoms	16	12	13	12	NS
Minor vascular comorbidity	78	81	66	84	<0.001
Socioeconomic disadvantage*	17	51	21	73	<0.001

* Percentage of patients in the lowest quintile of socioeconomic disadvantage.

NS indicates not significant (p > 0.10); COPD, chronic obstructive pulmonary disease; LRTI, lower respiratory tract infection; UTI, urinary tract infection.

Details of the multivariate regression models that extrapolated cost and survival to a lifetime horizon, as well as to estimate expected lifetime costs and survival, are presented in Additional file 1: Tables S1, S2, S3. The models include alternative sets of clinical and socio-demographic explanatory variables, indicating an effect of socioeconomic status that is additional to associations with poor health status. The survival curve plots indicate that the survival models were of good fit and produced sensible estimates (see Additional file 2: Figure S1 for the mean survival curve versus Kaplan-Meier survival curve plots for each intermediate endpoint). Table 2 presents observed, expected, and risk adjusted lifetime costs and survival (life-years), as well as the associated incremental costs per life year gained.

**Table 2:** Unadjusted and adjusted lifetime costs, survival, and incremental cost-effectiveness

**Hospital**	Observed*		**Incremental cost per LY gained**	**Expected**		**Standardized**^**†**^		**Incremental cost per LY gained**
	**Mean costs**	**Mean survival**		**Mean costs**	**Mean survival**	**Mean costs**	**Mean survival**	
B	$ 16,504	6.202	Dominated by hospitals A, C & D	$ 16,056	6.442	$ 449	−0.24	Dominated by hospitals A & D
C	$ 16,073	7.903	Dominated by hospital D	$ 15,244	8.084	$ 829	−0.18	Dominated by hospitals A & D
D	$ 12,471	8.139	Dominant	$ 16,855	8.088	-$ 4,384	0.05	-
A	$ 15,157	7.463	Dominated by hospital D	$ 14,626	7.098	$ 532	0.37	$ 15,632 (D vs. A)

Costs are reported in AUD. LY indicates life years.

*In line with the text, observed costs and survival incorporate predicted values beyond the observed intermediate endpoints, they are labeled as ‘observed’ values because they reflect the effects of the observed intermediate endpoints.

†Standardized costs and survival are estimated as observed minus expected values.

The unadjusted results show that Hospital D had the lowest mean costs and the highest mean survival, and therefore dominated the other hospitals.

The risk adjusted analysis shows that for both hospitals B and C, at least one other hospital had lower costs and higher survival (i.e. they were dominated). However, after risk adjustment, mean survival is greater for hospital A than for hospital D, though D remains the least costly institution. On average, patients treated at Hospital A incurred an additional cost of $15,632 to gain one additional life year, relative to Hospital D. Lowering the discount rates from 5% to 2% and 0% decreased the incremental cost per life year gained to $12,016 and $10,720, respectively.

**Figure 2:**
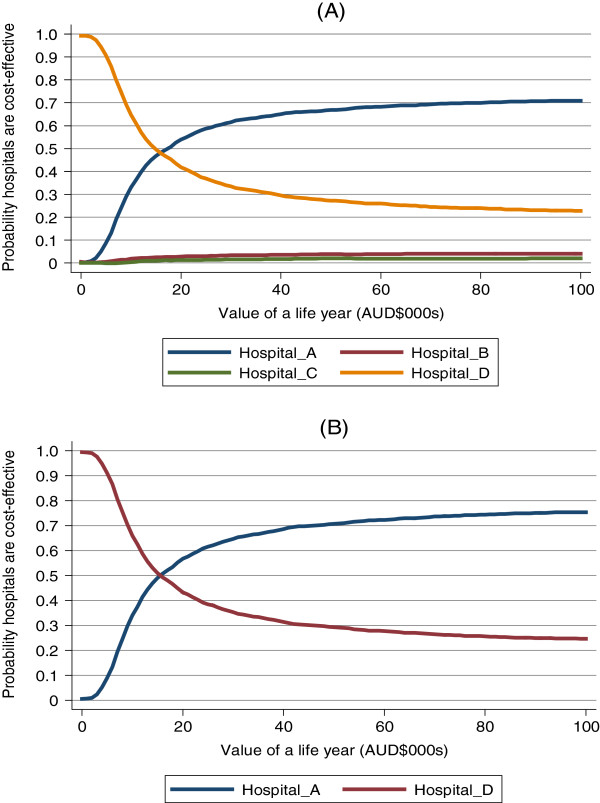
Cost-effectiveness acceptability curves for A) all 4 included hospitals and B) hospitals A & D only.

The results of the probabilistic sensitivity analysis are presented in the form of cost-effectiveness acceptability curves in Figure 2a, which shows the probability that each of the hospitals is the most cost-effective hospital at different monetary values for gaining life years. Hospital A has a 65% probability of being the most cost-effective hospital at a life year value of $50,000 – a commonly implied threshold. Comparing hospitals A and D directly (Figure 2b), hospital A had a 70% probability of being the most cost-effective hospital at a threshold of $ 50,000.

### Descriptive analyses

The RAC-E results provide an indication of the consequences of variation in clinical practice. The next stage is to investigate areas of variation and potential causes of the estimated differences in RAC-E. The following paragraphs describe some preliminary analyses of the available process data relating to the eligible patient cohort.

Table 3 presents available data on length of stay, and the use of rehabilitation services. The observed lower costs at hospital D may be mainly explained by the reduced LoS across all levels of stroke severity, though particularly for patients with catastrophic CC, for whom mean LoS is at least 10 days shorter at hospital D than at any other hospital. More patients at hospital D received sub-acute rehabilitation in each of the stroke categories, though the inter hospital differences were not statistically significant. With respect to LoS for sub-acute rehabilitation, it is noticeable that patients going onto rehabilitation from Hospitals B and C had significantly longer lengths of stay than patients at Hospitals A and D.

**Table 3:** Characteristics of stroke patients who did and did not have rehabilitation by stroke severity

	**Hospital A**	**Hospital B**	**Hospital C**	**Hospital D**	**P-value**
**B70A (STROKE + CCC)**					
**Rehabilitation:**					
Age (mean (sd), years)	79.6 (10.0)	76.4 (9.4)	74.4 (10.7)	76.2 (13.7)	NS
Proportion with rehab (%)	35	24	32	44	NS
Acute LoS (mean (sd), days)	18.3 (11.2)	23.9 (14.6)	18.3 (13.2)	8.1 (3.5)	<0.001
Rehab LoS (mean (sd), days)	23.6 (12.7)	39.4 (27.3)	42.0 (38.2)	24.4 (10.2)	<0.001
**No rehabilitation:**					
Age (mean (sd), years)	83.7 (7.0)	79.7 (8.5)	79.2 (9.4)	79.3 (12.6)	0.017
Acute LoS (mean (sd), days)	20.8 (15.0)	21.2 (12.1)	22.9 (20.7)	9.7 (6.8)	<0.001
**B70B (STROKE + SCC)**					
**Rehabilitation:**					
Age (mean (sd), years)	71.7 (10.5)	75.8 (11.5)	72.4 (11.3)	68.8 (12.2)	NS
Proportion with rehab (%)	45	47	45	54	NS
Acute LoS (mean (sd), days)	12.5 (7.8)	11.8 (8.2)	12.8 (5.8)	7.9 (4.2)	0.003
Rehab LoS (mean (sd), days)	23.9 (17.6)	34.4 (21.5)	41.6 (27.8)	25.4 (9.5)	<0.001
**No rehabilitation:**					
Age (mean (sd), years)	76.3 (12.0)	73.8 (10.4)	72.4 (14.8)	75.9 (12.1)	NS
Acute LoS (mean (sd), days)	13.4 (11.5)	13.3 (10.1)	10.1 (9.2)	8.3 (6.3)	0.028
**B70C (STROKE -CSCC)**					
**Rehabilitation:**					
Age (mean (sd), years)	72.1 (10.8)	71.0 (6.6)	70.3 (10.5)	66.6 (13.3)	NS
Proportion with rehab (%)	22	20	23	41	NS
Acute LoS (mean (sd), days)	9.9 (5.1)	8.0 (5.7)	9.5 (6.5)	8.7 (7.7)	NS
Rehab LoS (mean (sd), days)	23.9 (24.0)	38.4 (24.9)	22.7 (16.6)	25.2 (12.3)	NS
**No rehabilitation:**					
Age (mean (sd), years)	72.3 (11.5)	72.3 (12.2)	70.0 (13.7)	64.5 (17.7)	NS
Acute LoS (mean (sd), days)	5.8 (8.0)	8.5 (15.8)	6.0 (5.8)	3.8 (2.6)	<0.001

+CCC – catastrophic complication or comorbidity; +SCC - severe complication or comorbidity; -CSCC - neither catastrophic nor severe complication or comorbidity.

NS indicates not significant (p > 0.10); LoS, length of stay.

Table 4 presents disaggregated details of the available costing data. The cost differences correlate with the observed differences in LoS, with hospital D having the lowest costs in each category. Comparing the most cost-effective hospital A, with the dominated hospitals B and C, hospital A has consistently higher allied health costs, and lower imaging, pathology, and pharmacy costs. This may be indicative of alternative foci with respect to the management of acute stroke patients.

**Table 4:** Breakdown of Stroke costs by DRG for each hospital

COST CATEGORIES*	**Mean (SD) Costs**				
	**Hospital A**	**Hospital B**	**Hospital C**	**Hospital D**	**P-value**
**B70A (STROKE + CCC)**					
**Medical Ward**	817 (556)	1,436 (912)	1,335 (1,085)	747 (532)	<0.001
**Nursing Ward**	5,222 (4,595)	6,480 (4,994)	4,964 (4,628)	2,376 (2,243)	<0.001
**Allied Health**	1,406 (1,100)	799 (439)	951 (811)	362 (312)	<0.001
**Imaging**	437 (254)	483 (331)	556 (480)	277 (273)	<0.001
**Pathology**	251 (164)	534 (473)	627 (512)	180 (228)	<0.001
**Pharmacy**	381 (362)	489 (315)	389 (567)	208 (145)	0.006
**ICU**	63 (405)	145 (777)	492 (2,215)	100 (623)	NS
**B70B (STROKE + SCC)**					
**Medical Ward**	503 (355)	925 (736)	715 (638)	693 (487)	<0.001
**Nursing Ward**	2,461 (2,862)	3,356 (3,406)	2,365 (2,848)	1,588 (1,213)	0.006
**Allied Health**	994 (787)	532 (421)	555 (458)	238 (202)	<0.001
**Imaging**	380 (238)	419 (304)	381 (340)	240 (237)	0.005
**Pathology**	179 (235)	276 (241)	295 (192)	128 (114)	<0.001
**Pharmacy**	239 (254)	340 (283)	180 (290)	183 (115)	<0.001
**ICU**	440 (2,796)	152 (857)	100 (516)	30 (220)	NS
**B70C (STROKE -CSCC)**					
**Medical Ward**	337 (317)	476 (699)	396 (338)	462 (404)	NS
**Nursing Ward**	1,092 (1,134)	1,355 (2,255)	1,010 (1,301)	908 (894)	NS
**Allied Health**	504 (636)	240 (227)	235 (284)	114 (181)	<0.001
**Imaging**	318 (251)	376 (341)	407 (421)	229 (286)	0.039
**Pathology**	103 (65)	174 (184)	223 (156)	97 (137)	<0.001
**Pharmacy**	140 (166)	207 (289)	168 (430)	116 (94)	NS
**ICU**	30 (240)	0 (0)	40 (223)	37 (145)	NS

*Direct costs are provided only, does not include overhead costs. ICU indicates intensive care unit.

## Discussion

The results from this study indicate variation in risk adjusted lifetime costs and outcomes associated with acute stroke services at the four largest metropolitan hospitals in South Australia. The mean results imply that if patients currently treated at hospital D were to be treated at hospital A, we could gain life years at a cost of $15,632 per additional life year. If this is considered to be a cost-effective use of resources, the care pathways should be investigated with a view to disseminating practice at hospital A to the other hospitals.

The risk adjustment process was important as the unadjusted analysis showed hospital D to dominate all three other hospitals (i.e. to cost less and gain more), mainly due to the younger age of the patient cohort attending that hospital. Hospital D also had a catchment area that includes lower socioeconomic areas than the other hospitals, which was captured as an area level variable in the risk adjustment process. The use of area level socioeconomic data generally reduces the differences between the groups at the ends of the advantaged-disadvantaged spectrum [12], but as we are comparing mean values across multiple areas, the aggregated nature of the socioeconomic data is unlikely to have a significant effect on the RAC-E analysis. This is convenient as individual level socioeconomic variables are difficult to specify via routinely collected data sources.

We propose that RAC-E analyses be undertaken to identify areas of clinical practice in which there are important differences in costs and outcomes at alternative institutions. Where important differences are identified, a second stage comparative analysis of process may be undertaken to identify components of the applied clinical practice processes that may be contributing to the estimated differences in costs and outcomes.

This paper has identified important differences in costs and outcomes across the four hospitals, but only preliminary data relating to the applied clinical practice processes are currently available. Interestingly, of the three hospitals, only hospital D did not have a specialized stroke unit. The other main difference was between the services provided at Hospitals A, B, and C, which indicated greater use of allied health at the more effective Hospital A, with larger imaging, pathology, and pharmacy costs at the less effective Hospitals B and C.

More detailed data are available from routine data systems other than those accessed to inform the presented RAC-E analysis. These data will inform more detailed analyses of clinical practice in the future, including aspects such as the use of specific technologies, such as tissue plasminogen activator (tPA) and magnetic resonance imaging (MRI), as well as data describing the timing of key events along the process, such as time to tPA, or time to inpatient admission.

A previous observational cohort study, comparing costs and survival of stroke patients across Europe, also noted that spending more on stroke services did not necessarily improve outcomes [13], which is the case here for Hospitals B and C. Grieve et al. [13] also identified differences in process, finding that the type of staff input varied across centres: nursing input at a stroke unit in Florence was provided entirely by fully qualified nurses, whereas at a stroke unit in London, 40% of the nurses had only received a basic level of training.

### Limitations

The use of routinely collected data provides access to a large and representative sample of eligible patients, but is reliant on data recorded for purposes other than research. Perhaps the most significant limitation concerned the use of AR-DRG and ICD-10 diagnostic codes to represent stroke severity and presence of co-morbidities, which provides limited scope to differentiate between patients. The inclusion of socioeconomic variables may reflect some of the unobservable variation in casemix [14], but future RAC-E applications will investigate the use of alternative data sources that facilitate greater differentiation between patients, for example, defining the extent to which a diabetic co-morbidity is controlled by accessing pathology reports.

The quality of the diagnosis-related variables is subject to the accuracy with which relevant information is recorded in patient notes, and then extracted by data coders. However, the coded data informs a significant proportion of each hospital’s budget, providing common incentives to all hospitals. Central audits also provide assurance of coding quality, and more importantly the lack of systematic differences between the hospitals.

There are also uncertainties around the data linkage, though again there is no reason to suspect systematic bias in the linkage processes. Going forward, current investments in data linkage facilities across Australia will improve linkage processes, providing access to a broader range of health care data (e.g. covering primary and secondary settings), as well as placing a greater emphasis on improving data quality and recording practices.

The analysis included only data describing inpatient hospital admissions, linked to population-based mortality data. The exclusion of other hospital or community-based services will not affect the measurement of outcomes, but could misrepresent cost differences. We emphasize that the purpose of the reported analyses is not to make definite conclusions regarding the relative cost-effectiveness of services provided at different hospitals, but to inform further investigation of the underlying causes of the estimated differences in cost-effectiveness. Further investigations might assess differences in clinical pathways using improvement tools such as process mapping [15,16], or process mining [17], to describe patient journeys and gain a better understanding of the content, sequence, and timing of the health care provided at different hospitals, including activities such as post-discharge monitoring and referrals to community-based health programs.

## Conclusion

Despite the development of evidence-based clinical practice guidelines for the management of acute stroke, the uptake of guidelines has been variable. Translating evidence into practice is challenging, as it often requires investment at both the organisational and practitioner levels [18]. The presented RAC-E methodology quantifies the costs and effects of variation in alternative clinical practice processes. Combined with complementary process analyses that identify specific areas of variation in clinical practice, RAC-E analyses are hypothesized to provide a powerful tool to aid the dissemination and uptake of best clinical practice.

## Competing interests

The authors declare that they have no competing interests.

## Authors’ contributions

All authors have contributed significantly to the design and coordination of the study. CP, OC and JK performed the statistical analyses and interpretation of data and drafted the manuscript. All authors made substantial contributions to the critical revisions of the manuscript and gave final approval of the version to be published.

## Pre-publication history

The pre-publication history for this paper can be accessed here:

http://www.biomedcentral.com/1472-6963/12/266/prepub

## Supplementary Material

Additional file 1 **Table S1.** Relevant covariates for the stroke cost extrapolation models. PDF - **Table S2.** Relevant covariates for the stroke survival extrapolation models. PDF - **Table S3.** Relevant covariates for the expected lifetime survival and cost models. Click here for file

Additional file 2 **Figure S1.** Mean survival curves versus Kaplan-Meier survival curves for intermediate endpoints. Click here for file
