# Supplementary material for: Evaluating the effects of variation in clinical practice: a risk adjusted cost-effectiveness (RAC-E) analysis of acute stroke services
Source: BMC Health Serv Res. 2012 Aug 21;12:266. doi: 10.1186/1472-6963-12-266 (PMC3526450; doi:10.1186/1472-6963-12-266)
Supplement: Additional file 1 — Table S1. Relevant covariates for the stroke cost extrapolation models. PDF - Table S2. Relevant covariates for the stroke survival extrapolation models. PDF - Table S3. Relevant covariates for the expected lifetime survival and cost models. [file 1472-6963-12-266-S1.pdf]

ADDITIONAL FILE 1

Evaluating acute stroke services: risk adjusted cost-effectiveness (RAC-E) analysis using routinely collected data.

C Pham, O Caffrey, J Karnon, D Ben-Tovim, P Hakendorf, M Crotty.

Table 1. Relevant covariates for the stroke cost extrapolation models
Table 2. Relevant covariates for the stroke survival extrapolation models
Table 3. Relevant covariates for the expected lifetime survival and cost models

Table 1. Relevant covariates for the stroke cost extrapolation models

Explanatory variables	Recurrent Stroke Event, Year 1		Recurrent Stroke Event, Year 2		Major Cardiac Event, Year 1		Major Cardiac Event, Year 2		No event	
	Non-zero cost	Annual cost	Non-zero cost	Annual cost	Non-zero cost	Annual cost	Non-zero cost	Annual cost	Non-zero cost	Annual cost
Age	-0.030 (0.000)					0.199 (0.000)			-0.414 (0.001)	
Age^2		0.003 (0.000)							0.006 (0.001)	
Age^3		-0.000 (0.000)				-0.000 (0.000)		-0.000 (0.000)	-0.000 (0.000)	
Age x sex					0.064 (0.004)					
Age^3 x sex					-0.000 (0.008)					
Stroke severity		0.874 (0.000)	0.167 (0.036)							
Recurrent stroke severity		-0.482 (0.000)								
Sex		1.430 (0.000)	-0.358 (0.001)		-3.605 (0.002)					
Septicaemia		1.085 (0.000)		-1.326 (0.000)		-0.698 (0.014)				
Anaemia		-1.318 (0.000)				0.530 (0.003)		-0.346 (0.042)		
Dementia incl. Alzheimer's				-1.067 (0.001)			-0.417 (0.006)		-0.542 (0.000)	
Acute LRTI & influenza		0.489 (0.027)								
Chronic obstructive pulmonary disease		-0.682 (0.000)								
Urinary tract infection		-0.771 (0.000)				0.366 (0.033)			-0.175 (0.005)	
Urinary symptoms		0.557 (0.000)			-0.445 (0.002)					
Minor vascular co-morbidity		1.020 (0.000)						0.430 (0.005)		
Major vascular co-morbidity							0.422 (0.011)		0.259 (0.000)	
Vascular procedures								0.679 (0.000)		
Circulatory disorders with AMI								1.071 (0.000)		
Venous thrombosis					-1.337 (0.003)			0.528 (0.006)		
Infective endocarditis						-1.501 (0.000)				
Hypertension						-1.266 (0.000)		-1.102 (0.000)		
Non-major arrhythmia						-0.687 (0.000)				
Percutaneous coronary intervention							0.904 (0.029)			
Peripheral vascular disorders							0.323 (0.032)	0.668 (0.001)		
Coronary artherosclerosis							0.418 (0.001)			
Diabetic co-morbidity		-0.436 (0.000)	0.339 (0.003)						0.193 (0.000)	0.334 (0.005)
Renal co-morbidity		1.316 (0.000)								
Renal co-morbidity x hemiplegia	1.244 (0.000)									
Lower limb or decubitus ulcer				-0.697 (0.003)						
Hemiplegia		-0.379 (0.000)								
Amputation for circulatory system								-0.324 (0.001)		
Malignancy								0.574 (0.009)	-0.284 (0.013)	
Intestinal disorders									0.153 (0.005)	-0.258 (0.029)

(Continued)

Table 1. Continued

Explanatory variables	Recurrent Stroke Event, Year 1		Recurrent Stroke Event, Year 2		Major Cardiac Event, Year 1		Major Cardiac Event, Year 2		No event	
	Non-zero cost	Annual cost	Non-zero cost	Annual cost	Non-zero cost	Annual cost	Non-zero cost	Annual cost	Non-zero cost	Annual cost
SEIFA decile									0.025 (0.049)	
Economic resources decile	0.628 (0.000)								-0.032 (0.018)	
Socioeconomic disadvantage decile	-0.561 (0.001)	-0.145 (0.000)								
SEIFA x socioeconomic disadvantage deciles							0.028 (0.000)			
SEIFA x education and occupation deciles							0.023 (0.001)			
Socioeconomic disadvantage x education and occupation deciles							-0.052 (0.000)			
Public patient							-0.191 (0.038)			
Emergency patient		1.036 (0.000)								
Transferred patient		0.860 (0.004)								
Constant			-1.231 (0.000)	10790 (0.000)			-1.010 (0.000)	9.183 (0.000)	7.289 (0.013)	9.035 (0.000)

Values reported are coefficient values (p-values).

Logistic models predicted probabilities of patients incurring costs in a particular year, GLMs predicted the cost magnitude, if incurred.

SEIFA - aggregate socioeconomic index for areas variable; SEIFA, socioeconomic, economic resources, and education and occupation deciles: 1 (lowest socioeconomic status), 10 (highest socioeconomic status).

LRTI - lower respiratory tract infection.

AMI - acute myocardial infarction.

Table 2. Relevant covariates for the stroke survival extrapolation models

Explanatory variables	Recurrent Stroke Event	Major Cardiac Event	No event
Age^3		1.000 (0.000)	
Age(sqrt)			2.471 (0.000)
Age^2 x recurrent stroke severity	1.000 (0.000)		
Age x stroke severity			0.985 (0.001)
Age^2 x stroke severity			1.000 (0.004)
Age^2 x sex			0.999 (0.000)
Age x dementia incl. Alzheimer's			1.073 (0.000)
Age^2 x dementia incl. Alzheimer's			0.999 (0.000)
Stroke severity		0.761 (0.000)	
Recurrent stroke severity	0.286 (0.000)		
Sex		0.657 (0.000)	
Anaemia		1.423 (0.003)	1.354 (0.006)
Dementia incl. Alzheimer's		1.323 (0.000)	
Acute LRTI and influenza	2.128 (0.000)		
Chronic obstructive pulmonary disease	1.620 (0.012)	1.569 (0.000)	1.290 (0.034)
Urinary symptoms	1.724 (0.000)		
Minor vascular co-morbidity			1.272 (0.002)
Heart failure and shock		1.310 (0.019)	
Non-major arrhythmia		0.698 (0.017)	
Diabetic co-morbidity		1.323 (0.010)	
Renal co-morbidity x sex		1.384 (0.000)	
Renal co-morbidity x acute LRTI and influenza		1.673 (0.001)	
Malignancy	2.045 (0.000)	1.649 (0.002)	
Socioeconomic disadvantage decile			0.964 (0.004)
Emergency patient			1.885 (0.000)
_rcs1	2.516 (0.000)	2.697 (0.000)	2.324 (0.000)
_rcs2	1.097 (0.015)	1.083 (0.010)	
_rcs3	0.885 (0.000)	0.933 (0.000)	

Values reported are coefficient values (p-values).
Royston Parmar flexible survival models were fitted, _rcs (restricted cubic splines) indicate the number of inflexion points in the fitted curves.
Education and occupation decile: 1 (lowest socioeconomic status), 10 (highest socioeconomic status).
LRTI - lower respiratory tract infection.

Table 3. Relevant covariates for the expected lifetime survival and cost models

Explanatory variables	Lifetime survival	Lifetime costs
Age	0.506 (0.001)	-1.634 (0.000)
Age^2	1.015 (0.000)	
Age^3	0.999 (0.000)	
Age^2 x dementia incl. Alzheimer's	0.996 (0.000)	
Age^3 x dementia incl. Alzheimer's	1.000 (0.000)	
Stroke severity	0.786 (0.000)	-17.433 (0.000)
Sex	0.598 (0.000)	
Dementia incl. Alzheimer's	224369 (0.000)	
Acute LRTI and influenza		11.816 (0.030)
Chronic obstructive pulmonary disease	1.380 (0.018)	
Urinary symptoms		9.604 (0.022)
Minor vascular co-morbidity	1.208 (0.026)	
Major vascular co-morbidity		20.195 (0.000)
Diabetic co-morbidity		7.753 (0.022)
Stroke severity x renal co-morbidity	1.124 (0.004)	
Malignancy x anaemia	3.183 (0.000)	-39.092 (0.000)
Minor vascular co-morbidity x diabetic co-morbidity	1.273 (0.006)	
SEIFA decile	1.102 (0.012)	
Socioeconomic disadvantage decile	0.873 (0.000)	
Economic resources decile		-1.161 (0.016)
Hospital D		-21.081 (0.000)
Constant		267.300 (0.000)
_rcs1	10.723 (0.000)	
_rcs2	0.330 (0.000)	
_rcs3	0.262 (0.000)	
_rcs4	0.417 (0.000)	
_rcs5	0.760 (0.000)	

Values reported are coefficient values (p-values).
Royston Parmar flexible survival models were fitted, _rcs (restricted cubic splines) indicate the number of inflexion points in the fitted curves.
GLM predicted the cost magnitudes.
SEIFA - aggregate socioeconomic index for areas variable; SEIFA, socioeconomic, and economic resources deciles: 1 (lowest socioeconomic status), 10 (highest socioeconomic status).
LRTI - lower respiratory tract infection.
