# Supplementary material for: Evaluating the effects of variation in clinical practice: a risk adjusted cost-effectiveness (RAC-E) analysis of acute stroke services
Source: BMC Health Serv Res. 2012 Aug 21;12:266. doi: 10.1186/1472-6963-12-266 (PMC3526450; doi:10.1186/1472-6963-12-266)

ADDITIONAL FILE 2

Evaluating acute stroke services: risk adjusted cost-effectiveness (RAC-E) analysis using routinely collected data.

C Pham, O Caffrey, J Karnon, D Ben-Tovim, P Hakendorf, M Crotty.

Figure 1. Mean survival curves versus Kaplan-Meier survival curves for intermediate endpoints

Figure 1. Mean survival curves versus Kaplan-Meier survival curves for intermediate endpoints

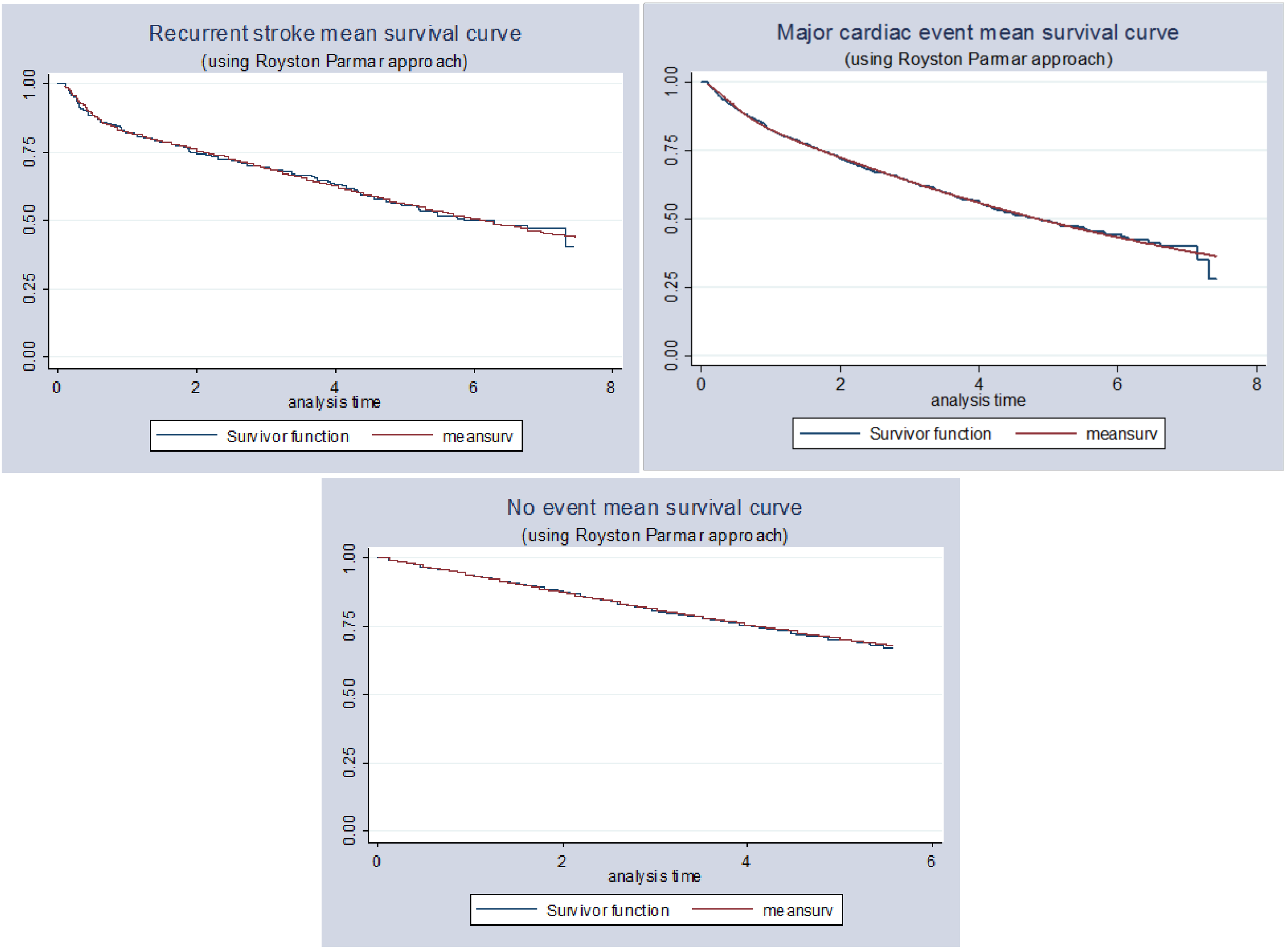

Supplement: Additional file 2 — Figure S1. Mean survival curves versus Kaplan-Meier survival curves for intermediate endpoints. [file 1472-6963-12-266-S2.pdf]
